# Experimental Study on Time-Dependent Changes in Rheological Properties and Flow Rate of 3D Concrete Printing Materials

**DOI:** 10.3390/ma14216278

**Published:** 2021-10-21

**Authors:** Hojae Lee, Eun-A Seo, Won-Woo Kim, Jae-Heum Moon

**Affiliations:** Department of Structural Engineering, Korea Institute of Civil Engineering and Building Technology Daehwa-Dong, Goyang-si 10223, Gyeonggi-do, Korea; sea0524@kict.re.kr (E.-A.S.); kimwonwoo@kict.re.kr (W.-W.K.); mjh4190@kict.re.kr (J.-H.M.)

**Keywords:** 3D concrete printing, ordinary Portland cement, shear stress, plastic viscosity, pumpability

## Abstract

Three-dimensional concrete printing (3DCP) materials require a relatively low water-to-binder ratio (W/B) of 0.3 or less to ensure their buildability and flow properties are sufficiently maintained after mixing. In this study, the rheological properties of 3DCP materials with W/B 0.28 were evaluated up to 60 min after mixing, and the yield stress and plastic viscosity were analyzed over time. A gradual decrease in flow rate with time was observed during the transport of 200 kg of material per batch through a 20 m hose. To examine the time-dependent changes in flow rate and layer volume, a 2200 mm × 1000 mm test specimen was printed. The dependence of the layer width over time during the printing process was measured and analyzed. The experimental analyses showed that the flow rate and layer volume of the 3DCP material gradually decreased with time after mixing, which was correlated with the rheological properties.

## 1. Introduction

Three-dimensional concrete printing (3DCP) technology has recently been employed in successful construction attempts, including a two-story building in the UAE by Apis Cor in 2019 and pedestrian bridges by TU Eindhoven in the Netherlands and Tsinghua University in China. These examples have demonstrated the tangible progress that 3DCP has undergone in the nearly 20 years since Contour Crafting was announced by the University of Southern California in the early 2000s [1]. The recent development of 3D printing technology has grown to encompass the areas of multi-story buildings, horizontal structural members, and other civil engineering applications, and the related success stories have been promoted in many public media outlets. As a result, 3DCP has been positioned as a promising future construction technology.

Current 3DCP processes commonly involve additive layering with material extrusion using cementitious binder-based (mainly OPC—ordinary Portland cement) cement paste, mortar, and concrete as the main materials as listed (Table 1). Because cementitious binders undergo setting and hardening with time after hydration, the material must retain a flowability appropriate for 3DCP for a sufficient pumpability until extrusion. Nevertheless, to ensure buildability of layers and printing member, the yield stress and plastic viscosity of the 3DCP material were increased in a previous study [2] because these properties aid in supporting the weight of successively added layers and maintaining the shape of the structure.

According to previous research, a low water-to-binder ratio (W/B) may decrease the slump that occurs immediately after mixing; however, a large slump loss still develops over time, indicating that the workability decreases with decreasing W/B [17]. Likewise, although the initial yield stress of a material may be high, it will also quickly begin to increase after mixing, which shortens the available open time for 3D printing [18]. Previous studies on the time-dependent changes in 3DCP materials have shown that the yield stress increases with time after mixing [2,15,19]. Materials for 3DCP are produced with relatively high yield stresses compared with conventional mortar and concrete to ensure buildability, and thus it stands to reason that the yield stress will increase to a greater degree over time after production.

**Figure 1:**
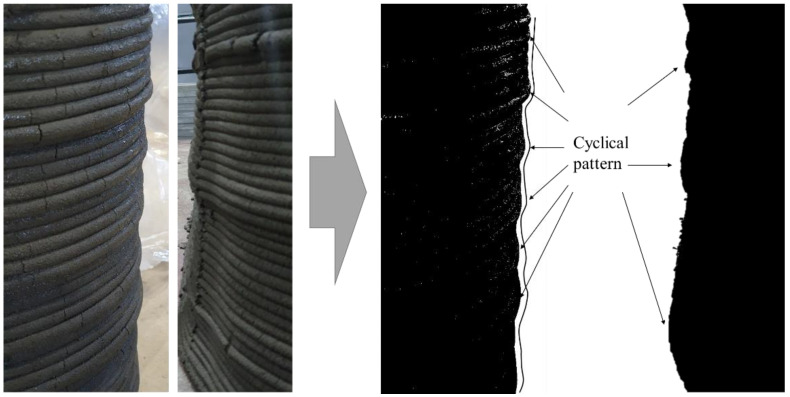
Appearance of cyclical pattern on printed layers.

**Figure 2:**
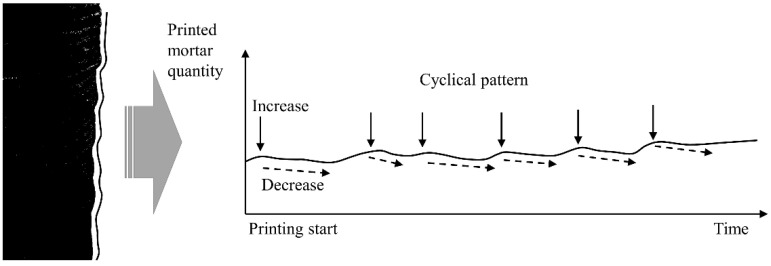
Cyclical pattern on printed layers over time in a real case.

Jang et al. [20] examined the effect of the properties of concrete on the pumping pressure and found that a high yield stress corresponds to a relatively large decrease in pressure during pumping. The materials used for 3DCP are transferred to a nozzle by pumping after mixing. If the yield stress and plastic viscosity increase during material transport, the feed amount will decrease under the same pumping pressure conditions. The cyclic pattern was observed in the process to measure the layer width on the surface of printed member during printing in the previous study [8] as shown in Figure 1. A cyclical pattern was observed in which the layer width initially decreased, abruptly increased after several layers, began to decrease again, and the cycle was repeated, as shown in Figure 2.

As shown in Figure 1 and Figure 2, the cyclical pattern on the actual printed member appeared even though the material was printed under the same material used and mixing ratio during the printing process. The printing time for one batch of 200 kg took 30–40 min, and it was confirmed that the width of the printed layer continued to decrease regardless of the printing time.

In this study, the main cause of the formation of the cyclical pattern was the decrease in the amount of material extrusion over time, and experimental verification was conducted to analyze the tendency of the amount of extrusion and change in cross-sectional area of the layer. To analyze the first factor, the extrusion amount of the material was analyzed using the same material and output system used in Figure 1 and Figure 2. To secure the reliability of the extrusion amount measurement, the extrusion amount was analyzed using nine batches of 200 kg units. In addition, to analyze the reducing trend of the cross-sectional area of the layer, the width of the layer was measured while the laminate was printed.

Cement-based materials undergo hydration with time from the moment they are in contact with water, and hydration affects the fluidity and rheological properties of these materials. In previous 3D printing-related studies, rheological properties were measured to predict the extrudability and buildability of materials [21]. In this study, the rheological properties were analyzed over time, and the correlation among experimentally measured flow rate, layer volume, and rheological properties was also analyzed.

## 2. Experimental Design for Pumpability Tests

### 2.1. Rheological Properties

The materials used for the experiments were OPC, fly ash (FA), silica fume (SF), a high water reduction agent (HWRA, liquid type, polycarboxylate agent), a viscosity modifying agent (VMA, powder type, cellulose), and sand (maximum aggregate size of 0.2 mm, single-particle-size silica sand). The mix proportions listed in Table 2 were designed to analyze the W/B dependence of the rheological properties of the materials at W/B of 0.28.

**Figure 3:**
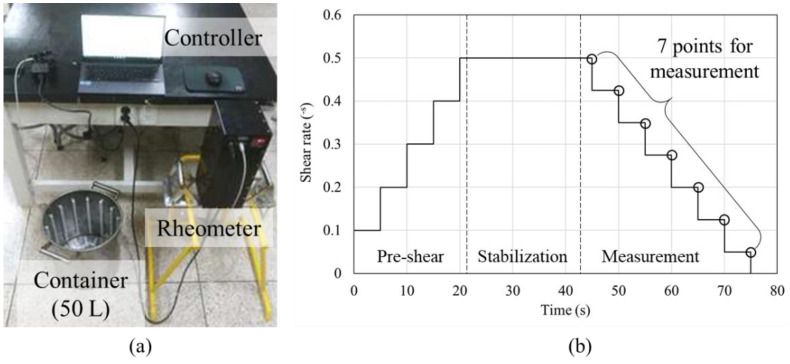
Rheometer and shear rate control program: (**a**) Rheometer; (**b**) share rate control program.

The materials used in this study were evaluated in terms of their yield stress and plastic viscosity depending on time using the concrete rheometer shown in Figure 3a. The rheological properties were measured after material mixing, after pumping (5 min after mixing), 30 min and 60 min. To measure the rheological properties, the shear rate was set over time according to the program shown in Figure 3b. To ensure reliable rheological measurements, the materials were homogenized in pre-shear and stabilization steps. In the pre-shear stage, the shear rate was increased in steps of 0.1 s^−1^ to a maximum of 0.5 s^−1^ over 20 s, after which the shear rate was maintained at 0.5 s^−1^ for another 20 s for the stabilization stage. After stabilization, the shear rate was reduced by 0.075 s^−1^ every 5 s, and a total of seven points were measured for yield stress and plastic viscosity analysis.

### 2.2. Preparation for Pumpability Tests

The pumpability tests were performed using mix proportion given in Table 1. OPC, FA, SF, sand, and VMA were premixed and prepacked to a total of 200 kg in a bag for each batch (excluding water and HWRA as liquid components) to ensure homogeneous mixing. Each batch was mixed for a total of 6 min after the addition of water, with one minute each of clockwise and counterclockwise rotation repeated three times. The mixed mortar was then immediately poured into the hopper of the pump.

**Figure 4:**
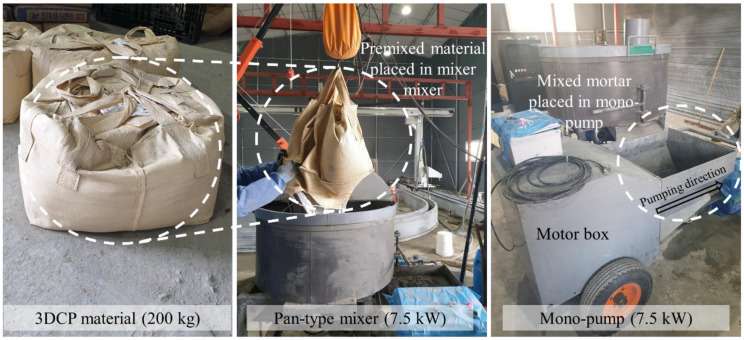
Premixed material, pan-type mixer, and mono-pump.

**Figure 5:**
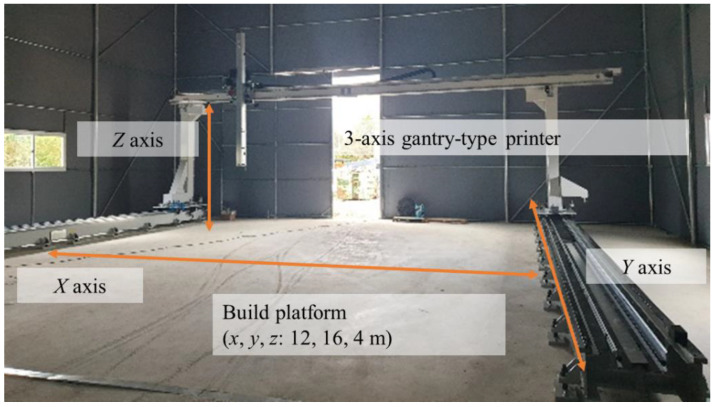
Gantry robot for 3DCP.

The equipment used in the experiments included a batch-type mixer, mono-pump, and 20 m long 1-wire reinforced hydraulic hose (Figure 4). For the mortar mixer, we used a 7.5 kW pan mixer, which can mix more than 150 L at a time. The pump had a 7.5 kW motor with a maximum pressure of 40 bar, maximum grain size of 6 mm, and an applicable rotor-stator. For the hose, two 1-wire reinforced high-pressure hoses were connected in units of 10 m. As shown in Figure 5, the apparatus used in the test was connected to the printing nozzle using a gantry robot with a workspace of 12 m × 16 m × 4 m (*w*·*l*·*h*).

### 2.3. Pumpability Test with Open Time

**Figure 6:**
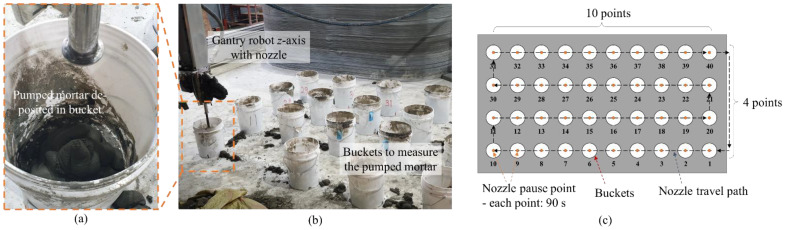
Weight and temperature measurement during pumping test: (**a**) pumped mortar deposited in bucket; (**b**) gantry robot on bucket; (**c**) nozzle travel path.

To evaluate the change in the 3DCP material feed rate with open time after mixing, the period during which each material batch was transferred from the pump after input of the mixture was uniformly set. For the pump, the rotational speed of the speed reducer was fixed so that this parameter had no effect on the amount of extruded material. For the pumpability test, 40 buckets were prepared to measure the material weight, and the gantry robot was set to measure the weight of the pumped material every 90 s, as shown in Figure 6. To measure material weight with time more precisely, nine batches were used for the pumping test (200 kg of premixed materials used for 1 batch).

### 2.4. Layer Shape Measurements

**Figure 7:**
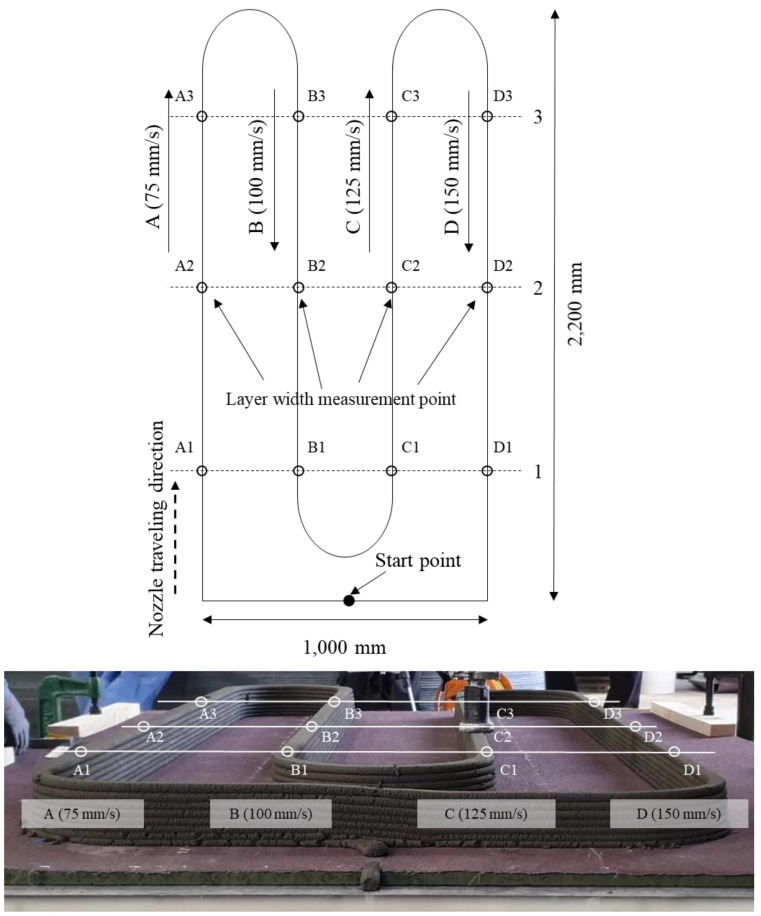
Printing design and measurement points.

A previous study confirmed that the layer volume of 3DCP materials gradually changes with the traveling speed of the nozzle and the printing time, but the cause of this change was not analyzed [8]. In this study, the change in the layer shape with printing time was measured to analyze its dependence on the volume of the printed material. The pump speed was fixed at 60 rpm, and the nozzle traveling speeds of 75, 100, 125, and 150 mm/s were used to examine whether it affected the layer width. The height of each layer was 10 mm, and the size of the printed specimen was set to a length of 2200 mm and width of 1000 mm. The overall shape was composed of four straight sections and three curved sections, as shown in Figure 7. The nozzle traveling speed was changed in the curved sections between each straight section. Approximately 89 s were required for the printing process to proceed from the indicated start point, through one complete printing cycle, and return to the starting point. One cycle was defined as the complete printing of one layer and return to the starting point. Measurements were taken over 21 cycles (approximately 1800 s). The layer shape was measured immediately after printing along the traveling path of the nozzle to ensure reliable measurements for each layer. As shown in Figure 7, three measurement points were set in each straight section following nozzle travelling speed (75, 100, 125, and 150 mm/s). 

## 3. Results and Discussion

### 3.1. Rheological Properties of 3DCP Materials

**Figure 8:**
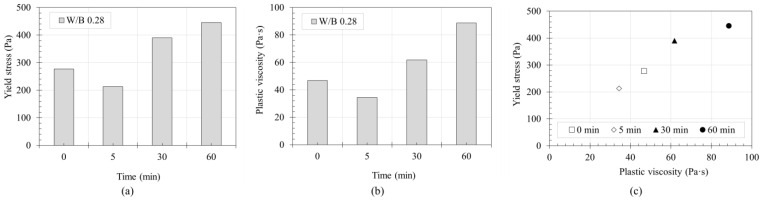
Evaluated rheological properties depending on time: (**a**) yield stress; (**b**) plastic viscosity; (**c**) relationship between plastic viscosity and yield stress.

The evaluated rheological properties of the 3DCP materials are shown in Figure 8. The experimentally measured properties are presented according to time flow for three cases: 0 min, 5 min, 30 min, and 60 min. The yield stress was measured to be 277.2 Pa immediately after mixing, which is approximately 5 times higher than the 40–50 Pa range of self-consolidating concrete [22]. Further, the plastic viscosity was measured to be 46.8 Pa∙s. 

The tendency of the yield stress and plastic viscosity of 3DCP materials to decrease depending on W/B has been observed in previous research [23,24]. The yield stress of material using there was approximately 400 Pa in the W/B range of 0.4–0.5. In the present study, however, the yield stress of approximately 400 Pa was measured at W/B = 0.28. This is considered to be due to the characteristics of the used constituent material such as HWRA, VMA, and aggregate size as well as the measurement method and device for rheological properties. The yield stress of the 3DCP materials in this study tended to increase after mixing continuously from 5 min, which is similar to the result of the previous study [25]. The experimental results in the previous study showed that the yield stress increased with the flocculation rate and thus with time. The above result was also supported by the plastic viscosity calculations, as shown in Figure 8b. The plastic viscosity of the material at 5 min was lower than that immediately after mixing (0 min). In these results, it is considered that the yield stress and plastic viscosity decreased due to pumping. It is presumed that the constituents of mortar were mixed and rearranged homogeneously during pumping and extrusion.

**Figure 9:**
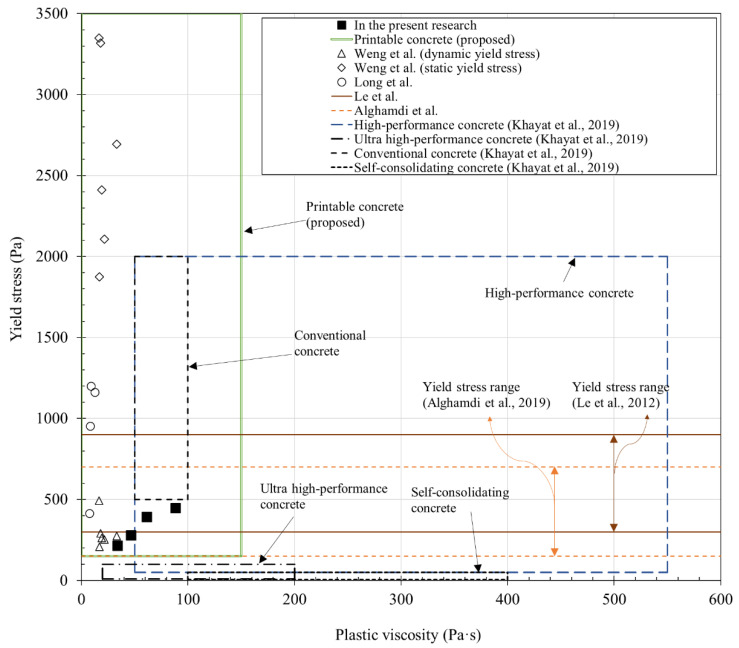
Rheological properties depending on concrete type [26,27,28,29].

Based on the results of Figure 8a,b, the relationship between yield stress and plastic viscosity over time is shown in Figure 8c. As shown, the yield stress continuously increased with the plastic viscosity from 5 min till 60 min. Considering the increasing trend of yield stress and plastic viscosity as shown in Figure 8, the weight per time unit of the 3DCP material used in this study is expected to decrease over time. This is in contrast to the rheological properties of 3DCP materials in a previous study [2], which retained their fluidity for up to 4 h or more. However, the flowability of these previously reported materials was maximized using a retarder. The rheological properties measured in this study are compared with previous results in Figure 9, and the properties of different concrete types are compared in Table 3. The yield stress and plastic viscosity ranges in Table 2 for different concrete types are also marked in Figure 9, which are found to be 213–445 Pa and 34–89 Pa∙s, respectively.

### 3.2. Flow Rate Change over Time

**Figure 10:**
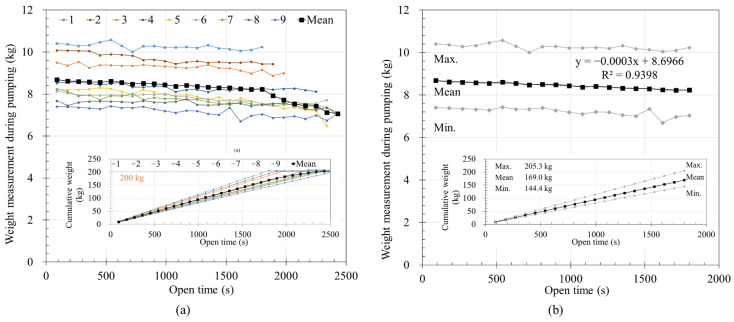
Pumpability test results: (**a**) measured weight of mortar during pumping and cumulative weight; (**b**) max. min. and mean value of mortar weight measured during pumping and cumulative weight (0–1800 s).

The measured mortar weights during continuous pumping immediately after mixing Figure 10. Figure 10a shows the results for the nine batches acquired every 90 s from the start of pumping. Experimental results are listed from batch 1 to 9 in the order in which extrusion was finished. As shown in Figure 10a, a batch that has a relatively high initial weight of extrusion was completely printed earlier than other batches. This was because the material, which was limited to about 200 kg per batch, was used faster. Therefore, as the amount of material pumped per unit time is relatively large, the time taken from the start to the end of pumping decreases.

The test of batch 1 was finished at 1800 s, therefore the average, maximum, and minimum measured weight per unit time of pumped mortar of all batches are summarized in Figure 10b using 1800 s. The results of the calculated average using the values from the beginning of pumping to 1800 s are plotted in black in Figure 10b. From the mean value in Figure 10b, the amount of pumped material can be predicted to decrease by 0.3 g/s from the pumping start, amounting to a total decrease of 540 g for 1800 s.

As shown in Figure 10a, the flow rate of each mix are in the range of 7–10.5 kg per 90 s. Although there is a change in the extrusion amount per unit time for each batch, not much difference is seen in the tendency of the extrusion amount to decrease in general. If the reason for the variation in extrusion per unit time for each batch is due to material characteristics, sand likely occupies the largest amount among the constituent materials. The premixing material used a dried material to prevent hydration during storage, and it can be inferred that the properties may have changed due to the contact between water and sand during mixing.

**Figure 11:**
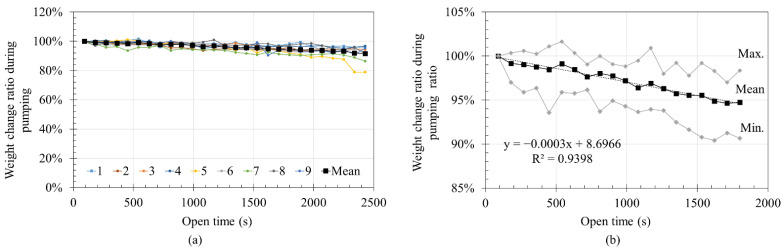
Weight decreasing ratio during pumping: (**a**) weight change ratio of pumped mortar; (**b**) mean weight change ratio and standard deviation of pumped mortar.

Figure 11a shows the weight reduction rate of the pumping material as a function of time. The amount of pumped material for each batch was set to 0% at the beginning of pumping as a reference point, and the changes in the weight of the pumped material were analyzed. The amount of pumped material repeatedly increased and decreased but generally tended to decrease over time. Similar to Figure 10b, Figure 11b shows the average weight change ratio and standard deviation of the batches up to 1800 s. The weight change ratio decreased by approximately 5% at 1800 s in Figure 11b. It could be predicted that the layer volume could gradually decrease with time under same printing condition. When pumping was continued until all material was discharged after mixing, the amount of extruded material tended to decrease with time, with a reduction of 5% depending on the time.

### 3.3. Layer Shape Measurements

**Figure 12:**
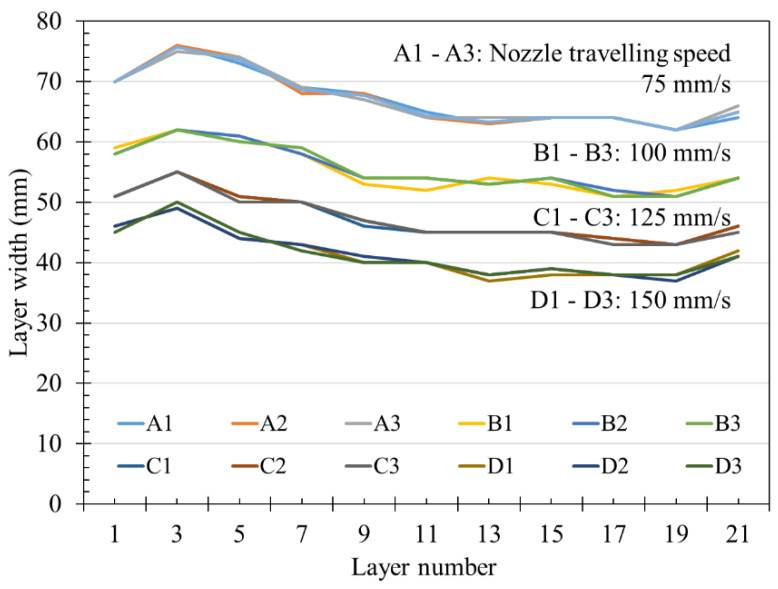
Dependence of layer width on printing cycle at the measured locations.

Figure 12 shows the continuously measured layer width. The measured layer width ranges for the different nozzle traveling speeds were 62–76 mm at 75 mm/s, 50–62 mm at 100 mm/s, 42–55 mm at 125 mm/s, and 36–50 mm at 150 mm/s. The results indicated that the layer width decreased with the nozzle traveling speed. Overall, at the same nozzle traveling speed, the layer width decreased with printing time. In particular, the layer width gradually decreased from the 3rd to the 19th layer. However, the layer width briefly increased again with printing after the 19th layer for all the nozzle traveling speeds before decreasing again. 

**Figure 13:**
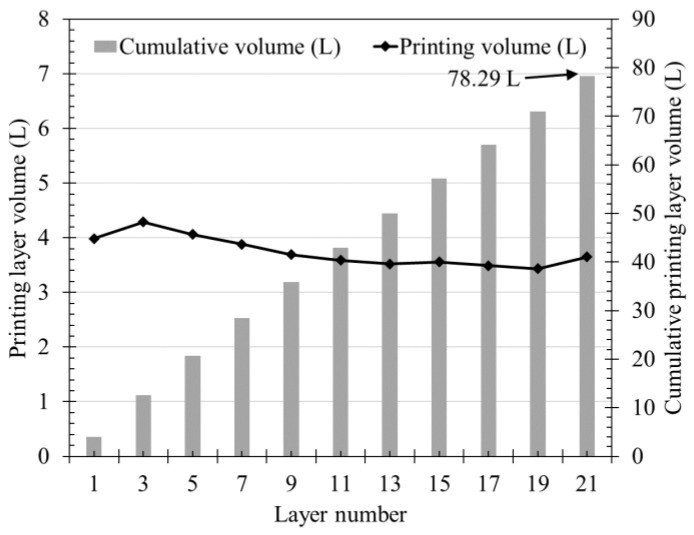
Volume of printed layer per cycle and cumulative printed layer volume.

In Figure 13, the volume of the layer assuming the shape of the layer is expressed as a rectangle. As for the width of the layer, the average width value of each layer was used from Figure 12, and the height of the layer was calculated as 10 mm, which is the actual power height; in addition, the cumulative printed layer volume over all the cycles is represented by a bar graph. The cumulative volume at 21 cycles, when the volume of the printed material began to decrease, was approximately 78 L. Assuming a mortar specific gravity of 2300 kg/m^3^, the weight was calculated to be 180 kg, which was included in the range of cumulative weight as shown in Figure 10b.

**Figure 14:**
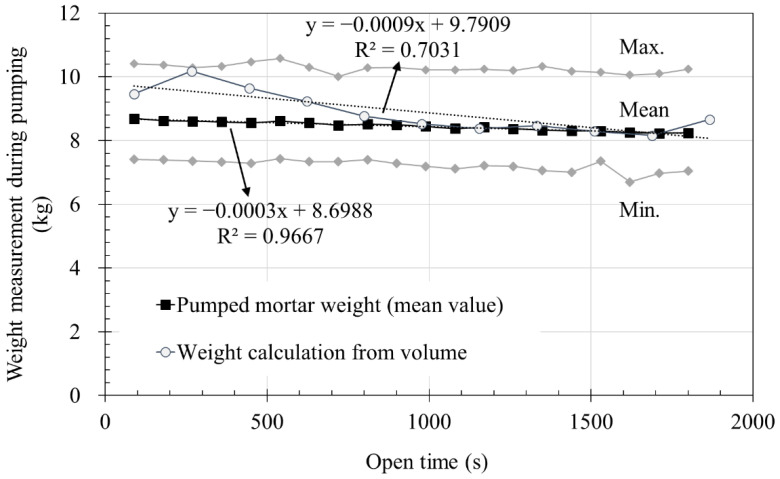
Measured weight of pumped mortar and weight calculated from layer volume.

In addition, the measured flow rate and the weight calculated from the measured layer volumes were compared, as shown in Figure 14. When the measured layer volumes over time were converted to weight using a specific gravity of 2300 kg/m^3^, the flow rate was determined to be approximately 8–10 kg/cycle. Taking into account the time for each cycle, the weight of the pumped material was the same as that corresponding to the measured layer volume. As a result, it can be inferred that the measured weights of the printed materials from the pump and the weights calculated from the measured printed layer volumes show similar trends with printing time.

### 3.4. Changes in Rheological Properties and Volume of Printed Layer

Airey et al. (2012) derived the pump power required for a mega-scale 3D printer through concrete fluid calculations [30]. As shown in Equation (1), the mean velocity was calculated assuming the volumetric flow rate (volume per unit time) required for the design. Based on the calculated mean velocity, as shown in Equation (2), the Reynolds number was calculated to derive the friction factor (Equation (3)), which was then used to derive the shear stress, as shown in Equation (4). By rearranging Equations (1)–(4) to give Equation (5), the volumetric flow rate can be derived from its relationship with the shear stress and plastic viscosity ratio.

(1) $$U_{m}=4Q/\pi D^{2}$$

(2) $$\;Re=\rho U_{m}D/\mu$$

(3) $$C_{f}=Re/16$$

(4) $$\tau =C_{f}\frac{1}{2}\rho U_{m}{}^{2}$$

(5) $$Q=\frac{\tau }{\mu }\left(\frac{\pi D^{3}}{36}\right)$$

Here, *U_m_* is the mean velocity of the extrusion material (m/s), *Q* is the volumetric flow rate (m^3^/s), *D* is the pipe diameter (m), *ρ* is the specific gravity (kg/m^3^), *μ* is the plastic viscosity (Pa·s), *C_f_* is the friction factor, and *τ* is the shear stress (Pa). As indicated by Equation (5), the flow rate is linearly proportional to the ratio of the yield stress and plastic viscosity.

**Figure 15:**
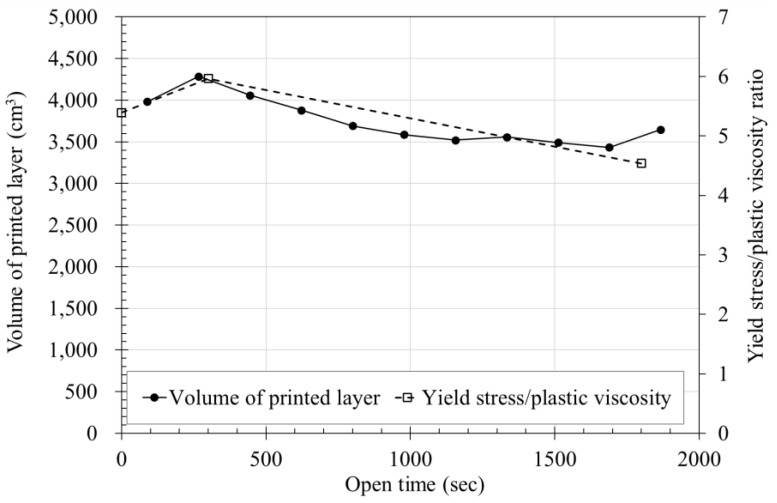
Volume and yield stress/plastic viscosity ratio of printed layers over time.

The results of yield stress and plastic viscosity shown in Section 3.3 were calculated according to Equation (5) and are plotted with time (by dotted lines) in Figure 15. In addition, the weight calculation results expressed in Figure 14 are also shown in Figure 15. The measured volume of printed layer showed a maximum of 4286 cm^3^ at about 270 s, and the measured yield stress/plastic viscosity ration showed a maximum of 5.97 at 300 s.

When the scale of the graph is adjusted based on the maximum value, it can be seen in Figure 15 that the trends of volume of printed layer and yield stress/plastic viscosity are similar. To derive the correlation between the two graphs, it is necessary to calculate the correlation coefficient, but in this study, there is a limit because the rheological properties and volume of the layer were measured using only one formulation. However, it is judged that the possibility of predicting the volume of the layer from rheological measurements is confirmed. Moreover, to predict this, the correlation between the rheology measurement result and layer volume must be derived through experiments and analysis of various cases.

## 4. Conclusions

In this study, we analyzed the cause of the change in the cross-sectional shape of laminated member using construction 3D printing technology. To analyze the cross-sectional shape change, rheology, material flow rate, and printed layer volume were measured over time using a mortar for 3D printing. The results are summarized as follows.

- The rheological properties that affect the flow rate tend to change with time. The yield stress and plastic viscosity continuously increased from immediately after mixing to 60 min and became almost twice. The yield stress increased from 213.4 Pa at 5 min up to 445.6 Pa at 60 min, and the plastic viscosity from 34.4 Pa∙s at 5 min up to 88.7 Pa∙s at 60 min. This confirms that the rheological properties are affected by time.
- A total of nine batches were used to measure the flow rate. As a result, it was confirmed that the extrusion amount decreased by about 0.3 g/s with time. At the start of extrusion, the measured amount was 8.69 kg, but after 1800 s, it became 8.24 kg. Considering that the unit measurement time is 90 s, the initial flow rate, which was 96.56 g/s, was reduced by about 5 g/s to 91.56 g/s at 1800 s. At 1800 s, the extrusion amount decreased by more than 5% to about 94.8% of the initial value.
- As for the measurement results of nine batches, a maximum of about 3 kg difference occurred in one measurement even under same material and extrusion conditions. This difference is expected to be due to the absorption rate of the aggregate based on the material properties alone. However, the decreasing trend of the extrusion amount with time showed a similar trend in all nine batches, thus it does not affect the flow characteristics of the material over time.
- The layer width was measured by printing 21 layers of a laminate having a length of 1000 mm, width of 2000 mm, and height of 10 mm. As a result of measuring the layer width, the layer width decreased as the number of layers increased. Through this, it was confirmed that the width of the layer decreased with time even under same conditions.
- Using the measured width of the layer, cross-sectional area, and volume of the layer, the weight of the output layer during one cycle were calculated, which was confirmed to be included in the range of flow rate results. The initial four cycles were higher than the average value of the flow rate measurement results, but after that, the results were almost similar, suggesting that the flow rate and the cross-sectional area of the layer are correlated.
- The volume of printed layer was compared using the measurement results of material yield stress and plastic viscosity, which affect the flow rate of the layer. It was confirmed that the two results showed similar trends. However, since the types of materials used in this study are limited for deriving an accurate correlation, additional research is needed.

In this study, the prediction method of printed layer volume was proposed using rheological properties as shown in Figure 15. The time-dependent changes in the rheological properties (yield stress/plastic viscosity ratio) of 3DCP materials were analyzed to derive a correlation between the weight of the printed material and the volume of the printed layer. As a result, we demonstrated that it is possible to predict the flow rate of a material using the ratio of the yield stress and plastic viscosity. Further research will be needed to derive a clearer correlation between rheology characteristics and extrusion volume for the accurate prediction of layer volume.

## Figures and Tables

**Table 1:** Mix type for 3D printing used previous studies.

W/B	Binder					Sand Size	Reference	Year
	OPC	FA	SF	Slag	Special Type			
0.16	○	-	○	-	-	-	Arunothayan et al. [3]	2019
0.24	○	-	○	-	-	0.6, 1.18 mm	Sanjayan et al. [4]	2021
0.25	○	○	-	-	Calcium aluminate cement	-	Bao et al. [5]	2018
0.26	○	○	○	-	-	-	Wang et al. [6]	2020
0.26	○	○	○	-	-	0.39 mm	Ma et al. [7]	2017
0.28	○	○	○	-	-	0.16–0.2 mm	Lee et al. [8]	2020
0.28	○	○	○	-	-	0–2 mm	Le et al. [2]	2012
0.28	○	○	-	-	Sulfoaluminate	0–0.3 mm	Zhu et al. [9]	2019
0.3	○	○	○	-	cement	0.06–8 mm	Mechtcherine et al. [10]	2019
0.31	○	○	-	○	-	-	Suntharalingam et al. [11]	2020
0.32	○	○	-	-	-	-	Rahul and Santhanam [12]	2020
0.32	○	○	○	-	-	0–2 mm	Rahul et al. [13]	2019
0.35	○	-	-	-	-	0–1.2 mm	Xu et al. [14]	2019
0.35	○	-	-	-	Calcium sulfoaluminate	0–2 mm	Khalil et al. [15]	2017
0.35	○	-	○	-	Nano clay	0–1 mm	Zhang et al. [16]	2019

○, used.

**Table 2:** Mix proportions for rheological property and pumping tests (Unit: kg/m^3^).

W/B	Water	OPC	FA	SF	Sand	HWRA	VMA
0.28	197.50	493.74	141.07	70.53	1408.00	7.05	0.79

**Table 3:** Typical yield stress and plastic viscosity ranges for different concrete mixtures (Adpated from Ref. [26]).

Rheological Parameter	Conventional Concrete	Self-Consolidating Concrete	High-Performance Concrete	Ultra High-Performance Concrete	3DCP Material in This Study
Yield stress (Pa)	500–2000	5–50	50–2000	10–100	213–445
Plastic viscosity (Pa∙s)	50–100	100–400	50–550	20–200	34–89
